# Gellan Gum Enhances the Quality of Egg-Based Yoghurt by Changing the Water Phase Distribution and Improving the Gel Texture

**DOI:** 10.3390/foods14020296

**Published:** 2025-01-17

**Authors:** Yuanyuan Zhang, Jianwei Zang, Shutong Liu, Bingxu Yan, Daobang Tang, Jiguang Chen, Dongwen Li, Dayong Peng, Zebo Liu, Zhongping Yin

**Affiliations:** 1 Jiangxi Key Laboratory of Natural Products and Functional Foods, College of Food Science and Engineering, Jiangxi Agricultural University, Nanchang 330045, China; zhangyyuan2022@163.com (Y.Z.); zangjv@yeah.net (J.Z.); l1261782397@126.com (S.L.); yanbingxuybx@163.com (B.Y.); chenjiguang@jxau.edu.cn (J.C.); lidongwen2929@163.com (D.L.); 2 Sericultural & Agri-Food Research Institute, Guangdong Academy of Agricultural Sciences/Key Laboratory of Functional Foods, Ministry of Agriculture and Rural Affairs/Guangdong Key Laboratory of Agricultural Product Processing, Guangzhou 510610, China; tdbang@163.com; 3 Jiangxi Provincial Key Laboratory of Improved Variety Breeding and Efficient Utilization of Native Tree Species, Key Laboratory of Chemical Utilization of Plant Resources of Nanchang, College of Chemistry &Materials, Jiangxi Agricultural University, Nanchang 330045, China; dayongpeng@163.com

**Keywords:** egg-based yoghurt, gellan gum, quality, texture, intermolecular force

## Abstract

Egg-based yoghurt (EBY) is a novel yoghurt fermented by lactic acid bacteria with high nutritional and health values, serving as a potential alternative to milk-based yoghurt. However, the hardness, adhesiveness, and water-holding capacity of egg-based yoghurt need to be further improved. In this study, the improvement in EBY quality by gellan gum and its underlying mechanism were investigated. The results showed that gellan gum significantly improved the quality of EBY (*p* < 0.05). Among the five concentration gradients tested, the EBY supplemented with 0.045% gellan gum exhibited the best quality with the highest sensory score of 83.57. With the increasing amount of gellan gum, hydrogen bonding interactions in the yoghurt significantly increased, while ionic bonding remained unchanged, but hydrophobic interactions and disulphide bonding gradually decreased. Low-field NMR assay results demonstrated that gellan gum significantly raised the amount of strongly bonded water while decreasing the amount of immobile water in the yoghurt. Confocal laser scanning microscopy revealed that EBY with 0.045% gellan gum had a better texture, whereas too much or too little gellan gum led to a coarser structure. In summary, gellan gum altered the water phase state and enhanced the water holding capacity through increased hydrogen bonding interactions, which consequently improved the yoghurt’s texture and sensory qualities. This study provides a reference for the development and application of EBY.

## 1. Introduction

Yoghurt is a noted lactobacillus-fermented food known for its unique texture and rich flavour and popular worldwide [1]. The nutritional and health benefits of yoghurt are widely recognized by consumers. For instance, yoghurt contains a large amount of beneficial bacteria that promote gastrointestinal peristalsis and regulate the ecological balance of intestinal flora [2]. In addition, yoghurt has been shown to enhance immune function, mitigate the side effects of antibiotics, alleviate symptoms of irritable bowel syndrome, lower cholesterol levels, and exhibit antibacterial and anticancer properties [3]. As a result, yoghurt production and sales are substantial, with promising development prospects.

Most yoghurts on the market are produced from cow’s milk and other animal milks through lactobacillus fermentation. Additionally, there are also some other high-quality animal and plant proteins which can be processed into yoghurt. In light of the present circumstances, the development of new yoghurts based on non-dairy milks represents a promising research direction [4]. Eggs are highly nutritious, containing high-quality proteins with an ideal amino acid composition, as well as essential polyunsaturated fatty acids (PUFAs), carotenoids, vitamins, and other functional components [5]. The gelling ability of egg proteins is comparable to that of milk proteins, suggesting that eggs could become a key material in yoghurt production, potentially replacing cow’s milk [6,7]. Our laboratory has developed a novel EBY using egg as the primary raw material. However, EBY currently still faces issues such as unsatisfactory texture, and therefore, further quality improvement is required.

Hydrocolloids serve as valuable quality improvers for gel-based foods, exhibiting superior processing characteristics. Their addition to yoghurt can enhance certain sensory acceptability aspects of the yoghurt and mitigate synergistic effects [8]. Zhao et al. [9] showed that trehalose has better water retention, emulsion stabilization and other functions, which can significantly improve the texture of yoghurt, enhance the appearance, texture, consistency, viscosity, and other properties. The research results by Wang et al. [10] indicate that the addition of hydrocolloids from brasenia schreberi to yoghurt can enhance water retention, viscosity, elasticity, and durability. Furthermore, this additive also promotes the proliferation of live lactic acid bacteria. Adding hydrocolloids to yoghurt can improve its taste, flavour, and sensory qualities, making it a significant research focus in the yoghurt industry [11]. Gellan gum is a linear anionic polysaccharide gel polymer with superior gelling properties, widely used in foods like puddings, jellies, beverages, and dairy products [12]. Ge et al. [13] found that gellan gum significantly improved the WHC, hardness, and rheological properties of yoghurt. Kong et al. [14] showed that adding gellan gum to soy yoghurt elevated the gel’s WHC and stabilized the gel network structure, resulting in more desirable sensory qualities. Although some studies have reported the use of gellan gum to improve the quality of milk gel products, to our knowledge, no studies have examined its use in EBY.

This study explored the effect of gellan gum on improving the quality of EBY and its underlying mechanism. First, the impact of gellan gum additive amount on the sensory quality, WHC, pH, and titrate acidity (TA) of EBY was investigated, and the optimal gellan gum additive amount was identified. Then, the yoghurt with the optimal additive amount was comprehensively characterized in terms of texture, rheology, microstructure, and intermolecular forces to elucidate the mechanism by which gellan gum enhances the quality of EBY. This study provides a reference for the development and application of new EBY.

## 2. Materials and Methods

### 2.1. Materials

Fresh eggs and sugar were purchased from a local supermarket (Nanchang, China). Starter culture (*Lactobacillus delbruckii* ssp. *bulgaricus* and *Streptococcus thermophilus*) strains were purchased from Health Probiotics Co., Ltd. (Suzhou, China), Gelatin, high acyl gellan gum, β-cyclodextrin, and diacetyltartaric acid were purchased from Henan Dengchun Industry Co Ltd. (Nanyang, China). Butter, anhydrous citric acid, and yoghurt starter were purchased from Meizhuan Foods Co., Ltd. (Suzhou, China), Zhejiang Yino Biotechnology Co., Ltd. (Jinhua, China), and Jingjing Sunshine Trading Co., Ltd. (Beijing, China), respectively. Other chemical reagents were purchased from Solarbio Technology Ltd. (Beijing, China).

### 2.2. Egg-Based Yoghurt Production Method

The EBY was processed according to the method described by Zang et al. [15]. Apart from the addition amount of gellan gum, all other processing parameters and methods were the same as those reported by Zang et al. In this study, five concentration gradients of gellan gum (0%, 0.025%, 0.045%, 0.065%, and 0.085%) were set. The processing method and parameters adopted are briefly described as follows: Whole egg liquid was separated from freshly washed and sanitized eggs. Purified water (1.8 times the volume of the egg liquid), sugar (10%), gellan gum (at the aforementioned five concentration gradients), β-cyclodextrin (0.05%), diacetyl tartaric acid (0.1%), and butter (2%) were added to the separated whole egg liquid, and the pH of the mixture was adjusted to 6.00. The mixture was heated for 40 min and then homogenized using a colloid mill. After homogenization, the starter culture (0.2%) was added to the mixture. Subsequently, the mixture was fermented at 45 °C for 10 h and then refrigerated for 12 h to obtain the egg-based yoghurt.

### 2.3. Measurement of pH

The determination of pH was carried out according to the method of Ikram et al. [16].

### 2.4. Measurement of Titrate Acidity (TA)

The TA of the yoghurt was determined in accordance with the methodology proposed by Dorokhov et al. [17], using phenolphthalein as an indicator solution and 0.1 mol/L sodium hydroxide as the titrant. A total of five grams of EBY was put into a conical flask, and titrated with a 0.1 mol/L sodium hydroxide standard solution until the mixture turned slightly red, and the red colour did not change for 5 s. The consumed volume of sodium hydroxide solution was recorded, which was used to calculate the TA of the samples.

### 2.5. Measurement of Water Holding Capacity (WHC)

The WHC was determined using the method of Utami et al. [18] with some modifications. A total of 20 g of sample was placed in a 50 mL centrifuge tube and then centrifuged at 3000 rpm for 30 min at room temperature. The supernatant was discarded, and the mass of the remaining precipitate was weighed to calculate the WHC. The WHC calculation formula used is as follows:

$$WHC=\frac{M_{2}-M_{0}}{M_{1}-M_{0}}\times 100\%$$

where M_2_ is the total mass of the sample and the centrifuge tube after the supernatant removal; M_1_ is the total mass of the initial sample and the centrifuge tube; and M_0_ is the mass of the centrifuge tube.

### 2.6. Determination of Textural Properties

The textural properties of egg-based yoghurt were determined according to the method of Xi et al. [19] with some modifications, using a TA-XT plus texture analyser (Stable Micro Systems, Surrey, UK) with a cylindrical probe (P/0.5, R = 6.35 mm). The parameters were set as following: temperature, 25 °C; pre-test speed, 2.00 mm/s; mid-test speed, 2.00 mm/s; post-test speed, 2.00 mm/s; maximum strain, 50%; trigger force, 5.00 g. Three replications were performed for each sample.

### 2.7. Measurement of Rheological Properties

Referring to the method of Quan et al. [20] with some modifications, rheological properties were measured using a shear rate-controlled rheometer (Discovery Hybrid rheometer, TA Instrument, Taunton, MA, USA). Measurements of sample viscosity were performed under steady-state shear conditions. The main detection parameters were set as follows: temperature, 25 °C; shear rate range, 0.1 to 1000 s^−1^. Oscillation time sweep measurements were carried out at 1 Hz to determine the strain in the linear viscoelastic region of each sample. Oscillatory tests were then performed within the linear viscoelastic region. The strain was set to 2.45%, and the frequency was set from 0.01 Hz to 100 Hz. The change in storage modulus (G′) and loss modulus (G″) with frequency was automatically recorded.

### 2.8. Confocal Laser Scanning Microscopy Detection

Nile red and Nile blue dyes were used in the confocal laser scanning microscopy detection (CLSM), adopting the approach of Lei et al. [21] with some modifications. The two dyes were added to the yoghurt sample and then mixed thoroughly. The mixed sample was left at 4 °C for one hour to allow the fluorescent dyes to diffuse fully. The stained sample was placed on a slide with groove and observed under a confocal laser scanning inverted microscope (Olympus FV3000, Tokyo, Japan).

### 2.9. Low-Field Pulsed Nuclear Magnetic Resonance Detection

Low-field pulsed nuclear magnetic resonance detection (LF-NMR) was conducted according to the procedure of Xue et al. [22] with some modifications. In total, one millilitre of egg-based yoghurt was put into a 1.5ml NMR glass tube, and then the NMR probe was inserted for detection. The spin relaxation time (T_2_) was determined using CPMG sequences on a low-field pulsed NMR analyser (Niumag Co., Ltd., Suzhou, China). Detection conditions: SW, 200 KHs; TW, 2000.000 ms; TE, 0.2 ms; RFD, 0.02 ms. T_2_ times were calculated using the T_2_-cpmg curve fitting procedure.

### 2.10. Detection of Intermolecular Forces

The intermolecular forces were determined according to the method of selective protein solubility reported by Zhang et al. [23]. Two grams of EBY was taken and homogenized after adding 0.6 M NaCl to break the ionic bonds. Afterwards, the supernatant was taken by centrifugation. The protein content of the supernatant was determined by the bis-urea method using bovine serum protein as a standard. The centrifuged precipitate was then resuspended with 1.5 M urea, 8 M urea, and 0.5 M β-mercaptoethanol (β-ME), sequentially and separately, and homogenized and centrifuged as above to disrupt the hydrogen, hydrophobic, and disulfide bonds in the gel system. The protein content of the obtained supernatant on each occasion was determined in order to calculate the proportion of hydrogen, hydrophobic, and disulfide bonds in the gel system.

### 2.11. Sensory Evaluation

The sensory evaluation team consisted of 14 members, both male and female, who have received professional sensory evaluation training with adequate evaluation skills and were familiar with the quality characteristics of yoghurt. The sensory evaluation involved four sub-indicators, namely, appearance, aroma, texture, and taste. The scoring of each individual index was carried out separately. The overall sensory quality of each sample was expressed as the sum of the four individual scores. The scoring criteria are shown in Table 1, with a full score of 100. The final sensory evaluation score of each sample is expressed as means ± standard deviation. An analysis of variance (ANOVA) and Duncan’s new multiple range test were used to compare means at the 5% significance level using IBM SPSS Statistics version 26.0 (Armonk, NY, USA).

### 2.12. Statistical Analysis

Three replicates were set up for each experimental treatment, and each sample was tested three times in parallel. The results were expressed as mean ± standard deviation. Statistical analyses were conducted using IBM SPSS Statistics version 26.0 (Armonk, NY, USA). A one-way analysis of variance (ANOVA) was carried out, and means were compared using Duncan’s multiple range tests (*p* < 0.05).

## 3. Results

### 3.1. Effect of Gellan Gum on the Sensory Quality

**Figure 1:**
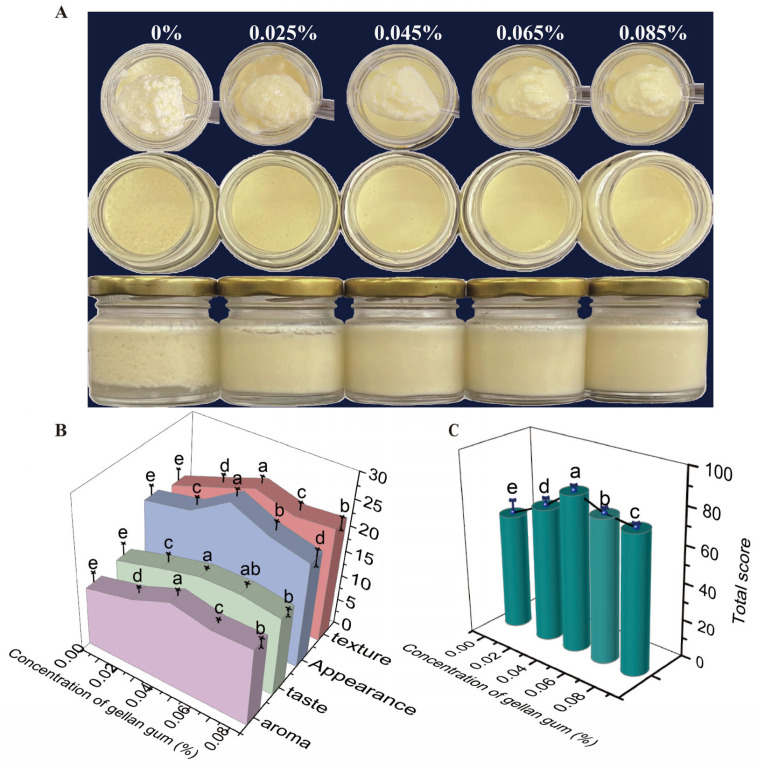
Effect of gellan gum on the sensory quality of egg-based yoghurt. (**A**): Appearance and morphology of egg-based yoghurt with different additive amounts of gellan gum. (**B**): Individual sensory evaluation results of egg-based yoghurt with different additive amounts of gellan gum. (**C**): Total sensory evaluation scores of egg-based yoghurts with different additive amounts of gellan gum. Different letters indicate means with significant difference at *p* < 0.05.

This study investigated the effect of gellan gum on the sensory quality of EBY, with an additive amount ranging from 0% to 0.085%. As illustrated in Figure 1A, EBY without gellan gum experienced dehydration and shrinkage, with a small amount of water precipitating at the bottom. Its structure was relatively rough, featuring several holes and slightly weaker gel strength. Consequently, yoghurt without gellan gum had the lowest sensory scores for appearance, aroma, texture, and taste (Figure 1B), with a total sensory evaluation score of only 61.92 (Figure 1C). As the additive amount of gellan gum increased, the EBY gradually became finer and smoother, and its gel strength progressively improved. The optimal sensory qualities for appearance, aroma, texture, and taste were achieved with a 0.045% additive amount of gellan gum, scoring 25.86, 16.71, 24.27, and 16.50, respectively, with a total score of 83.57. However, exceeding the 0.045% additive amount resulted in increased gel strength, leading to a jelly-like texture and some astringency, which caused the sensory score to decline. This decline might be attributed to the enhanced strength of the yoghurt gel network due to the gellan gum [24], resulting in changes in appearance and flavour. In summary, the optimal amount of gellan gum for EBY is 0.045%. To further elucidate the mechanism by which gellan gum improves the quality of EBY, the yoghurt was subsequently characterized and analysed in terms of texture, rheology, microstructure, water phase state, and intermolecular forces.

### 3.2. Effect of Gellan Gum on pH and TA

**Figure 2:**
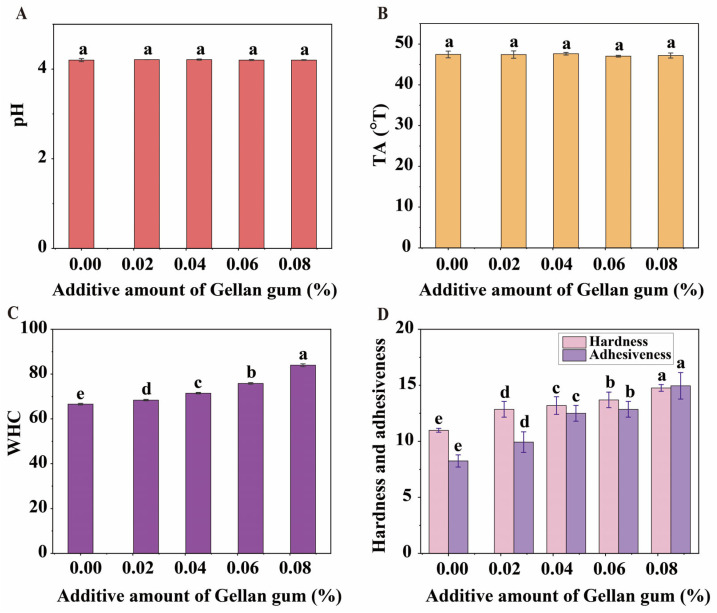
Effect of gellan gum additive amounts on pH (**A**), acidity (**B**), water holding capacity (**C**), and texture (**D**) of egg-based yoghurt. Different letters (a–e) indicate statistically significant differences (*p* < 0.05, Duncan’s new multiple range test) between samples.

The pH and TA of yoghurt are critical indicators of proper fermentation and acid production. Changes in pH can significantly affect the charge of proteins. When the pH approaches the protein’s isoelectric point, the protein undergoes acid coagulation, forming a gel and contributing to the yoghurt’s unique texture [25]. Therefore, pH and TA are important quality indicators for yoghurt. Figure 2A,B show that within the additive amount range of 0% to 0.085%, gellan gum had no significant effect on the pH and TA of the final yoghurt product. The pH of the yoghurt remained approximately 4.2, and the TA was around 47.5 °T. These results indicate that the additive amount of gellan gum does not adversely affect the fermentation and acid production by lactic acid bacteria.

### 3.3. Effect of Gellan Gum on the WHC

Yoghurt is a food with a high-water content. The WHC reflects its ability to retain internal moisture, making it an important indicator for evaluating yoghurt quality [26]. The results of the WHC test (Figure 2C) show that as the additive amount of gellan gum increased from 0% to 0.085%, the WHC of the yoghurt exhibited a continuous upward trend. Compared to the control group without gellan gum, the WHC of yoghurt with 0.085% gellan gum increased by 30.35%. This indicates that the additive amount of gellan gum significantly enhances the WHC of the product, which may be a crucial factor in improving the quality of EBY. This study further analysed the mechanism by which gellan gum improves the WHC of yoghurt from various perspectives, including water phase distribution, microstructure, and intermolecular forces.

### 3.4. Textural Characterization of Egg-Based Yoghurt with Different Gellan Gum Additive Amounts

The results of the textural tests showed that the additive amount of gellan gum significantly increased the hardness and adhesiveness of the EBY (Figure 2D). With 0.085% gellan gum, the hardness increased from 10.98 to 13.76 g and the adhesiveness from 8.25 to 14.95 g.sec, representing increases of 25.32% and 80.21%, respectively, compared to the control without gellan gum. Given the weak gel strength of EBY without gellan gum, the appropriate additive amount of gellan gum can significantly enhance the texture and mouthfeel of the product. However, excessive additive amounts can make the product too hard and sticky, negatively impacting its sensory quality. These textural results are consistent with the earlier sensory evaluation results, corroborating each other.

### 3.5. Changes in Water Phase Distribution in Egg-Based Yoghurt with Different Gellan Gum Additive Amounts

**Figure 3:**
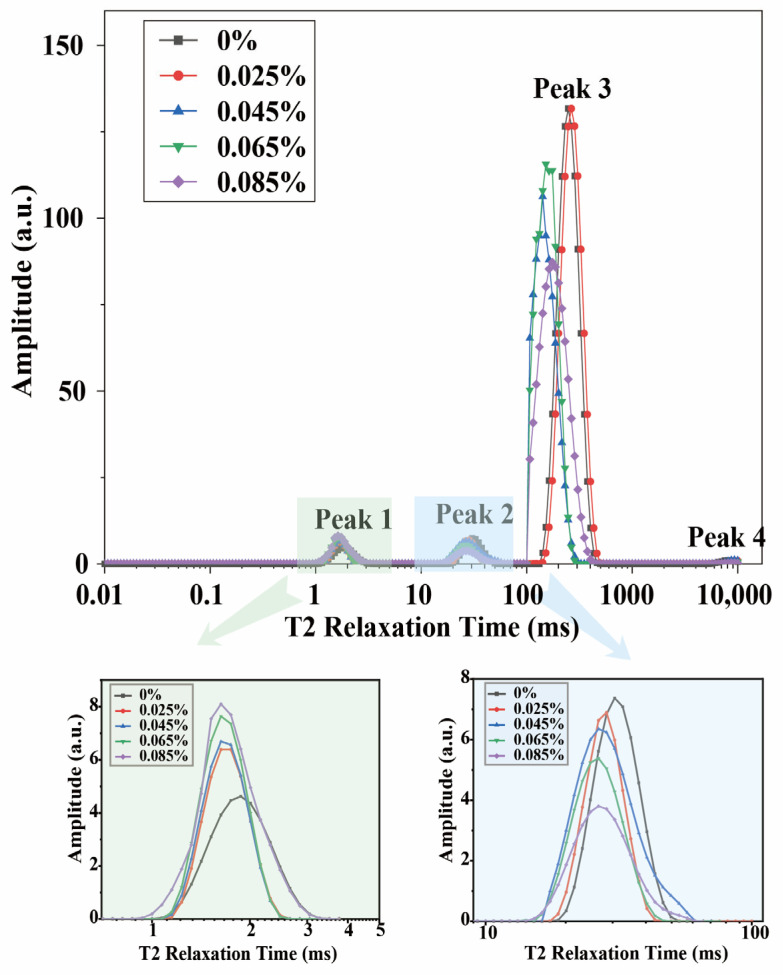
Results of relaxation time T2 of egg-based yoghurt with different amounts of gellan gum added by low-field magnetic resonance detection (low-field NMR). The values presented are representative graphs.

LF-NMR was used to characterize the changes in the moisture phase distribution of EBY at different gellan gum additive amounts. The T_2_ relaxation time reflects the distribution of water phases in yoghurt gels, providing a foundation for their quality and texture analysis [26]. The LF-NMR results (Figure 3) indicated the presence of four water phases in EBY: strongly bound water with a relaxation time of T_21_ (0.5–4.0 ms), weakly bound water with a relaxation time of T_22_ (10–60 ms), immobile water with T_23_ (100–700 ms), and free water with T_24_ (4000–10,000 ms). As the additive amount of gellan gum increased, the relaxation peak representing strongly bound water tended to shift to the left, with peak height and peak area gradually increasing. Conversely, the relaxation peak representing bound water also shifted to the left, but its peak height and peak area gradually decreased. These results indicate that the water phase state in EBY with gellan gum was significantly enhanced, with an increased proportion of strongly bound water and a decreased proportion of immobile water compared to the control group. This finding clearly explains why the WHC of the product significantly increased with the additive amounts of gellan gum and provides a basis for the improvement in the quality of yoghurt by gellan gum.

### 3.6. Microstructure of Egg-Based Yoghurt with Different Gellan Gum Additive Amounts

**Figure 4:**
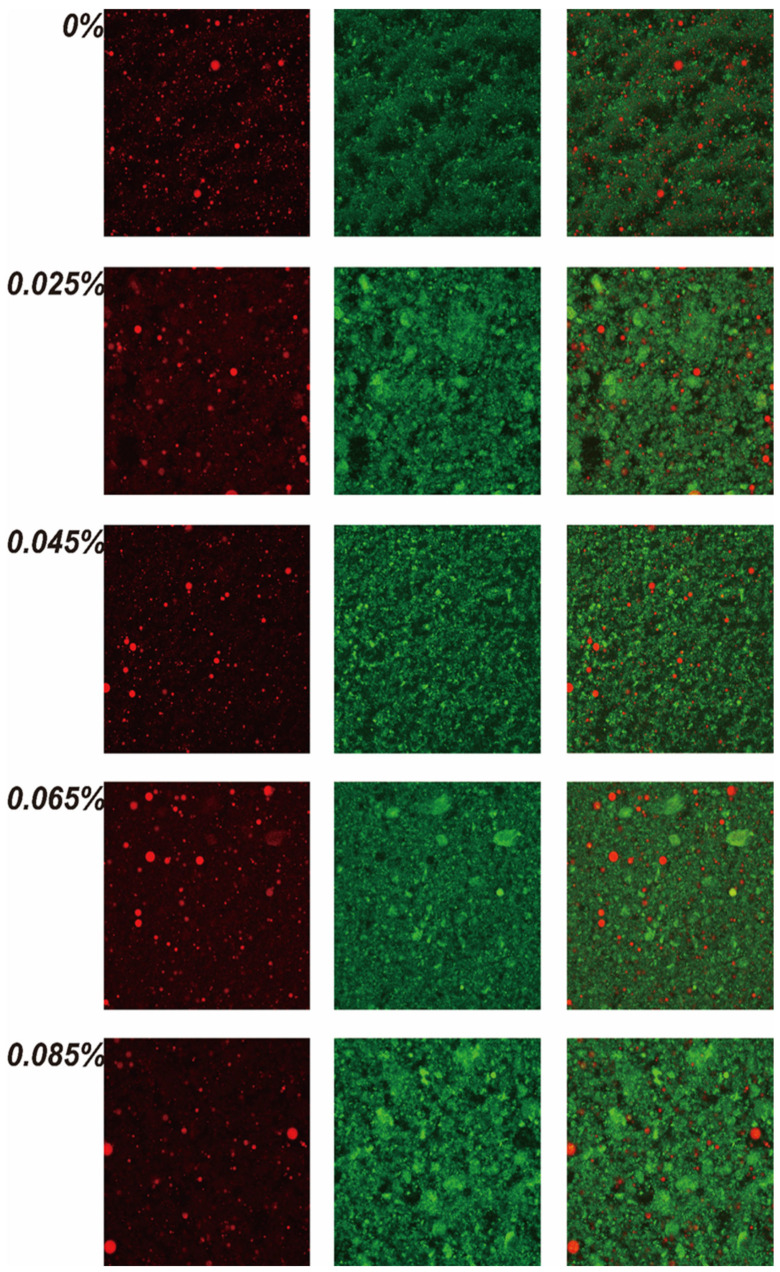
Images of egg-based with different gellan gum additive amounts by confocal laser scanning microscopy detection (CLSM).

The microstructure of EBY at different gellan gum additive amounts was characterized using CLSM. In the images, green represents the proteins in the gel stained by Nile blue, while red represents the lipid components stained by Nile red. According to the CLSM results (Figure 4), adding gellan gum does not alter the distribution of lipid components but significantly improves the gel’s microstructure. The EBY without gellan gum did not exhibit noticeable graininess, but numerous holes and grooves were visible in the gel. As the gellan gum additive amounts increased from 0 to 0.045%, the gel network structure became smoother, and the holes and grooves gradually decreased in size and number. However, when the gellan gum additive amounts continued to increase beyond 0.045%, the gel network structure became rough and uneven again, with more prominent holes and grooves. In conclusion, gellan gum significantly improves the microstructure of EBY gels, with 0.045% being the optimal additive amount, corroborating the results of the sensory evaluations.

### 3.7. Rheological Properties of Egg-Based Yoghurt with Different Gellan Gum Additive Amounts

#### 3.7.1. Effect of Gellan Gum on the Static Rheological Properties

**Figure 5:**
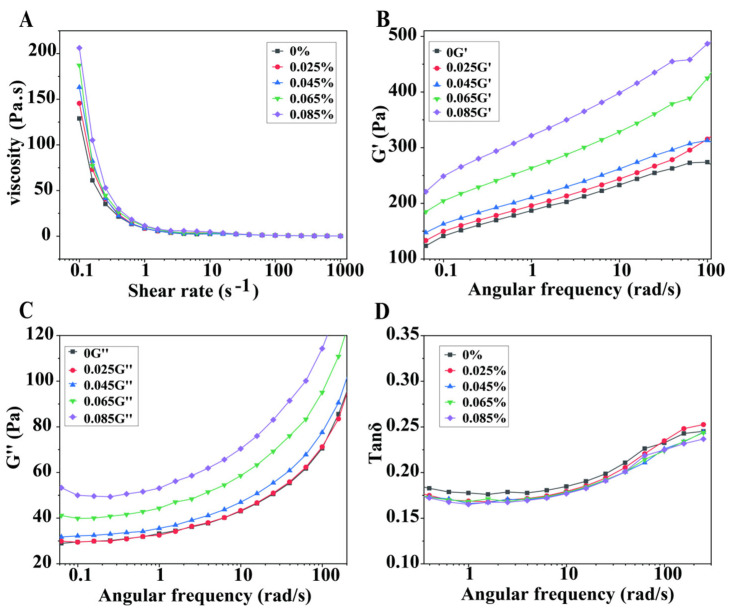
Rheological characterization of egg-based yoghurt with different gellan gum additive amounts. (**A**): Viscosity at different shear rates; (**B**): storage modulus at different angular frequencies (G′); (**C**): loss modulus at different angular frequencies (G″); (**D**): Tanδ at different angular frequencies. The values presented are representative graphs.

Figure 5A shows the viscosity of EBY with different concentrations of gellan gum at various shear rates. The figure indicates that EBY exhibits typical shear-thinning properties. Lazaridou et al. [27] also obtained the same results and conclusions on the rheological properties of yoghurt. As the concentration of gellan gum increases, the viscosity of EBY increases at low shear rates. This is mainly due to the thickening effect of gellan gum; as its concentration increases, the number of polysaccharide molecules per unit volume rises, and intermolecular entanglement increases, leading to higher viscosity. When the shear rate exceeds 10 s^−1^, there is no significant difference in viscosity at the gellan gum concentrations set in this study.

#### 3.7.2. Effect of Gellan Gum on the Dynamic Modulus Properties

Figure 5B,C illustrate the changes in storage modulus G′ and loss modulus G″ during strain sweep of EBY with different concentrations of gellan gum at a constant frequency of 1 Hz. As the concentration of gellan gum increases, the values of G′ and G″ also increase. G′ is consistently larger than G″, indicating obvious solid properties and elastic behaviour. These results suggest that higher concentrations of gellan gum led to a stronger yoghurt gel system. The phase angle (δ) analysis results in Figure 5D show that Tanδ is less than 1 for all control and test groups, indicating that EBY exhibits solid-like characteristics, similarly to the findings reported by Vénica et al. [28] for yoghurt. Increasing the gellan gum concentration results in a decrease in Tanδ, indicating that the additive amount of gellan gum enhances the solid properties of the yoghurt system, which corroborates the textural test results mentioned earlier.

### 3.8. Differential Characterization of Intermolecular Forces in Egg-Based Yoghurt with Different Gellan Gum Additive Amounts

**Figure 6:**
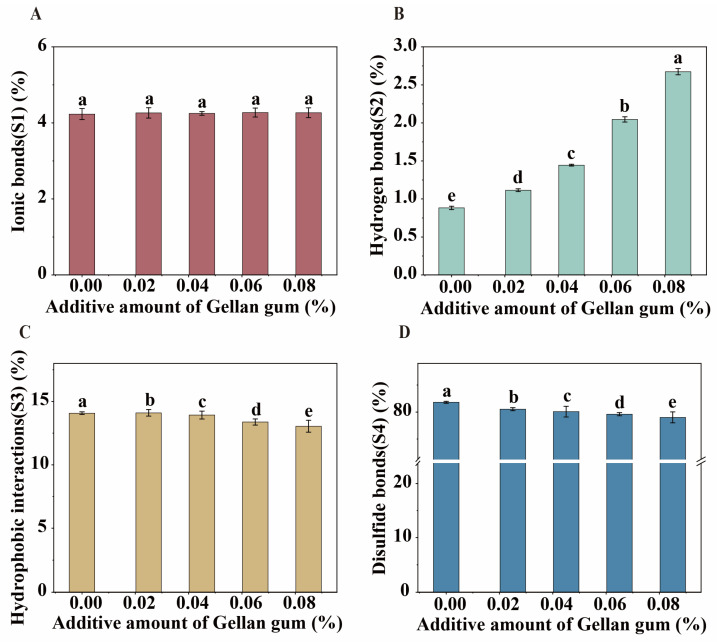
Variation in the ratio of intermolecular forces in egg-based yoghurt with different amounts of gellan gum addition. (**A**): Ionic bonding ratios of egg-based yoghurts at different gellan gum additions; (**B**): hydrogen bonding ratios of egg-based yoghurts at different gellan gum additions; (**C**): hydrophobic interactions ratios of egg-based yoghurts at different gellan gum additions; (**D**): disulfide bonding ratios of egg-based yoghurts at different gellan gum additions. Different letters (a–e) indicate statistically significant differences (*p* < 0.05, Duncan’s new multiple range test) between samples.

In this study, different denaturants were used to treat the EBY gel system to sequentially disrupt four different types of intermolecular forces: ionic bonding, hydrogen bonding, hydrophobic interactions, and disulfide bonding. This approach aimed to determine the roles these forces play in the formation of protein gels. The test results (as shown in Figure 6) indicated that when 0.085% of gellan gum was added to the EBY, the proportion of hydrogen bonding significantly increased from 0.88% to 2.66%, while the proportions of hydrophobic interactions and disulfide bonding slightly decreased from 14.08% and 80.81% to 13.04% and 79.99%. Ionic bonding did not show any significant change. This could be because gellan gum is a hydrophilic colloid, and as its concentration in the EBY increases, it forms more and stronger hydrogen bonding interactions within the system. This results in a more robust yoghurt system, enhancing its WHC and increasing the proportion of bound water. These findings are consistent with the previous results regarding WHC and LF-NMR analysis.

## 4. Discussion

Yoghurt has become very popular worldwide due to its high-quality protein content, unique texture, and flavour [29]. Although traditional yoghurt made from animal milk has high nutritional and health value, it also poses problems such as lactose intolerance and allergies [30]. Despite the availability of a few plant-based yoghurts made from grains, pseudo-grains, pulses, nuts, etc., these products have not changed the dominance of animal milk yoghurts [31].

Poultry eggs are rich in nutrients, including proteins and lipids, and exhibit excellent acid gelation capacity, making them theoretically ideal for yoghurt production [4,15,32]. Abundant egg resources make the large-scale production of poultry EBY possible [33]. However, there are no yoghurts on the market that use poultry eggs as the primary ingredient. Research on the application of eggs in yoghurt processing is limited [34], with most studies focusing on adding eggs to cow’s milk, but not exceeding 50%, thus still classifying these products as milk-based yoghurts. Therefore, the processing technology and principles of poultry EBY need urgent exploration.

Gellan gum is a microbial exopolysaccharide produced by *Sphingomonas elodea* and *Shingomonas paucimobilis*, consisting of a tetrasaccharide unit of two β-D-glucose residues, one β-D-glucuronic acid, and one α-L-rhamnose [35]. Studies have shown that gellan gum has good thermal and acid stability, adjustable gel elasticity and rigidity, high transparency, and good flavour release, exhibiting excellent functional properties in both solution and gel states [36]. Thus, it has a wide range of applications in the food industry. Li et al. [37] demonstrated that gellan gum exhibits remarkable gelling capabilities, forming gels with a robust network structure at low concentrations, acidic conditions, and elevated temperatures, making it suitable for enhancing the texture and structure of protein gels. Yulin et al. [37] found that gellan gum significantly improved 14 properties associated with pea isolate protein gels, including gel strength, WHC, and rheological properties, promoting the formation of a dense and homogeneous gel network structure. Zhou et al. [38] reported that the additive amount of gellan gum to potato protein gels improves gel strength, increases fracture stress and strain, and enhances elasticity and ductility.

In this study, gellan gum was utilized to enhance the quality of egg-based yoghurt. When the addition level of gellan gum was 0.045%, the egg-based yoghurt exhibited ideal quality characteristics, achieving the highest sensory score of 83.57. A texture analysis revealed that further increasing the gellan gum content led to a harder and stickier product, potentially due to overly strong complexation between the gellan gum and proteins [39], which resulted in a decline in sensory quality. As the gellan gum content increased from 0% to 0.085%, the water holding capacity of the yoghurt gel improved, with a significant increase in hydrogen bonding. The G′ and G″ values of the system also increased. This phenomenon can be attributed to the crosslinking of gellan gum molecular chains with proteins, locking water within a dense network structure [40], and the formation of more hydrogen bonds between hydroxyl groups in the gellan gum and hydrogen atoms in the water [41]. Low-field nuclear magnetic resonance (LF-NMR) results indicated that gellan gum significantly increased the amount of bound water and reduced the amount of immobilized water in the yoghurt, which explains the observed changes in water-holding capacity and intermolecular forces. Confocal laser scanning microscopy (CLSM) images showed that the egg-based yoghurt with 0.045% gellan gum had a finer texture, while yoghurt with other gellan gum concentrations had relatively coarser structures. This is because the appropriate addition of gellan gum can improve texture, but further increases in the gellan gum concentration may be detrimental to the gel structure [42]. It can be inferred that the addition of an appropriate amount of gellan gum alters the water phase in the yoghurt gel, and enhances the product’s hardness and viscosity by increasing hydrogen bonding interactions, thereby improving its water-holding capacity, texture, and sensory quality.

In addition to gellan gum, many other hydrophilic colloids can be used in food applications, such as carrageenan, guar gum, acacia gum, pectin, and chitosan. These hydrocolloids have different physicochemical and processing properties, especially gelation properties, providing various options for developing and improving gel foods. Skryplonek et al. [43] developed a high-quality, lactose-free frozen yoghurt using *κ*-carrageenan as a stabilizer. Jiang et al. [44] investigated the effect of pectin on yoghurt texture and stability, analysing the appearance and taste, and found that adding pectin increased the gel-breakage strength and distance, enhancing the hardness, density, and viscosity of stirred yoghurt, as well as significantly improving WHC and sensory acceptability. Mudgil et al. [45] reported that adding gelatin to camel milk yoghurt significantly improved whey synthesis and centrifugal separation, enhancing the structural and rheological properties of camel milk yoghurt. Due to time and effort constraints, this paper only investigated the effect and mechanism of gellan gum in improving the quality of EBY. Various hydrophilic colloids with different properties can be subsequently used to improve the quality of EBY for more desirable results.

## 5. Conclusions

In this work, gellan gum was employed to enhance the quality of egg-based yoghurt (EBY). The effects of gellan gum concentration on the quality of EBY and its underlying mechanisms were investigated through physicochemical, textural, rheological, microstructural, and intermolecular force characterizations. The study found that when gellan gum was added at a level of 0.045%, EBY achieved the best sensory score, reaching 83.57. However, further increases in the addition amount led to excessively high hardness and adhesiveness, giving the yoghurt a jelly-like texture, which negatively impacted the sensory quality. Based on this, various detection processes and characterizations were conducted, including physicochemical analysis, textural evaluation, rheological testing, microstructural observation, and intermolecular force measurements. The experimental results indicated that gellan gum significantly improved the water-holding capacity (WHC) of the gel system, increased the proportion of strongly bound water, and decreased the proportion of immobilized water, ultimately leading to corresponding changes in the rheological properties, microstructure, and texture of the yoghurt. Currently, there are no relevant reports on the use of gellan gum in the processing research and production applications of egg-based yoghurt. This study provides a reference for the theoretical and technical research on the processing of egg-based yoghurt.

## Figures and Tables

**Table 1:** Criteria for sensory evaluation of egg-based yoghurt.

Item	Indicator	Score
Appearance (with a full score of 30 points)	Uniform texture and colour, no air holes, slightly yellow, no water analysis.	22.5–30
	The texture and colour are relatively uniform, with a few air holes and no obvious water analysis.	15–22.5
	The texture is slightly rough, with obvious air holes and water analysis.	7.5–15
	The texture is uneven and rough, with many and large pores, and water analyses are evident.	0–7.5
Aroma (with a full score of 20 points)	It has the characteristic aroma of yoghurt, with a strong flavour and no off-flavour.	15–20
	It has a yoghurt taste and no off-flavour.	10–15
	Yoghurt flavour but slightly muted, slightly eggy, no discernible off-flavour.	5–10
	Egg-like or distinctly offensive odour.	0–5
Texture (with a full score of 30 points)	The texture is homogeneous and fine, with ideal hardness, viscosity, and elasticity.	22.5–30
	The texture is uniform and consistent, with moderate hardness, viscosity, and elasticity.	15–22.5
	Weak gels, too much or too little hardness, viscosity, and elasticity.	7.5–15
	No gel is formed or a hard gel is formed.	0–7.5
Taste (with a full score of 20 points)	It has the characteristic delicate and smooth texture of yoghurt, with a rich flavour and moderate sweetness and sourness.	15–20
	Delicate texture with the flavour that yoghurt should have.	10–15
	The texture is not subtle enough and the flavour is not rich.	5–10
	The palate is rough and grainy, with inappropriate acidity and sweetness.	0–5

## Data Availability

The original contributions presented in the study are included in the article, further inquiries can be directed to the corresponding authors.
